# Recognition of Juvenile Ankylosing Spondylitis in an Adolescent Female Presenting to a Chiropractor

**DOI:** 10.7759/cureus.100551

**Published:** 2026-01-01

**Authors:** Robert J Trager, Kelsey L Lewis, Cliff Tao, Roberto E Borgia

**Affiliations:** 1 Chiropractic, Connor Whole Health, University Hospitals Cleveland Medical Center, Cleveland, USA; 2 Family Medicine and Community Health, Case Western Reserve University School of Medicine, Cleveland, USA; 3 Chiropractic Radiology, Private Practice, Irvine, USA; 4 Pediatric Allergy, Immunology, and Rheumatology, University Hospitals Rainbow Babies and Children's Hospital, Cleveland, USA

**Keywords:** arthritis, axial spondyloarthritis, chiropractic, differential diagnosis, magnetic resonance imaging, sacroiliitis

## Abstract

Juvenile ankylosing spondylitis, a form of axial spondyloarthritis (axSpA), is an uncommon cause of chronic back pain in adolescents. We describe a 15-year-old female with a three-year history of chronic lumbosacral and hip pain, prolonged morning stiffness, nocturnal pain, and limited response to multiple conservative treatments, including physical therapy, medication, and injections. Previous lumbar magnetic resonance imaging (MRI) was virtually normal. On chiropractic evaluation, the combination of symptoms, chronicity despite conservative care, and lack of explanatory structural changes in the lumbar spine imaging raised suspicion for inflammatory sacroiliitis, leading to ordering of a pelvic MRI. This demonstrated bilateral structural lesions of the sacroiliac joints. Accordingly, the chiropractor prepared a referral to pediatric rheumatology, where the patient was diagnosed with juvenile ankylosing spondylitis and initiated on etanercept therapy with subsequent clinical improvement. This case underscores the challenges of diagnosing axSpA in adolescents and highlights the role of chiropractors as portal-of-entry clinicians in recognizing its signs and symptoms, pursuing appropriate imaging, and making timely referral when indicated.

## Introduction

Axial spondyloarthritis (axSpA) is a chronic, immune-mediated inflammatory disease affecting the sacroiliac joints and spine, resulting in pain, stiffness, and potentially irreversible structural damage if not recognized and treated promptly [1]. While axSpA is fairly uncommon, affecting 0.35% to 1.30% of the general United States population, it is even less common in the pediatric population [2]. Specifically, axSpA most often begins in the third or fourth decade of life, with 10-20% of patients experiencing symptom onset in childhood or adolescence [2].

Given the potential seriousness of the condition and the risk of progressive structural changes, delays in diagnosis remain a substantial problem. A systematic review focusing on adult axSpA found median diagnostic delays ranging from two to six years, with some cases exceeding eight years [3]. Studies have reported that an onset of symptoms before age 16 years, lack of radiographic features of axSpA, and negative human leukocyte antigen B27 (HLA-B27) are associated with an increased diagnostic delay [3,4]. A cohort study of 795 individuals, mostly young to middle-aged adults with chronic low back pain, found an axSpA prevalence of 5.8%, with about one in five cases previously undiagnosed, suggesting a substantial burden of unrecognized disease [5].

Chiropractors frequently evaluate patients with persistent low back pain and function as portal-of-entry clinicians in the United States, which is the setting of the current case [6]. Their role includes differentiating benign musculoskeletal causes of back pain from potentially serious conditions and initiating appropriate imaging or specialty referrals [7]. While there are a handful of cases focused on chiropractic management of axSpA in adults, previous review articles on the topic and a recent observational study suggest there are no published cases of chiropractors contributing to the diagnosis of axSpA in pediatric patients [8-10]. Early recognition of axSpA has the potential to reduce diagnostic delays and prevent pain and loss of function.

This study describes a 15-year-old female with a three-year history of low back and hip pain who was ultimately diagnosed with sacroiliitis consistent with the juvenile-onset ankylosing spondylitis form of axSpA after visiting a chiropractor and undergoing pelvic magnetic resonance imaging (MRI).

## Case presentation

In 2025, a 15-year-old female presented with her mother to a chiropractor within an extensive integrated healthcare system for evaluation of chronic low back pain and hip pain, and to explore additional diagnostic options. She described bilateral lumbosacral and upper gluteal pain of moderate-to-severe intensity, up to 7/10 on a numeric pain rating scale. Pain began insidiously three years prior and worsened after a minor skiing injury one year prior, and was now present daily. Although the patient had previously participated in several sports, including soccer and track, she was now limited in her ability to exercise. She reported lower back stiffness and pain lasting 1-2 h in the morning and occasional nocturnal pain waking her from sleep.

The patient had no pertinent medical history, no gastrointestinal symptoms, no vision problems, and no rash, although she had acne, which was treated with isotretinoin approximately two years ago. While there was no family history of spondyloarthropathies, her mother had a history of endometriosis. Over the preceding three years, the patient had sought care from multiple specialists, including her pediatrician, sports medicine, physical medicine and rehabilitation, pediatric orthopedics, pediatric rheumatology, and a different chiropractor who had performed spinal manipulation over a year earlier. She also underwent two courses of physical therapy focusing on hip and core strengthening, completed home exercise programs, practiced yoga, and underwent ultrasound-guided steroid injections into the iliopsoas tendon sheath for bilateral snapping hip. She had also trialed gabapentin up to 100 mg twice daily without relief and used over-the-counter analgesics with minimal benefit. Radiographs and magnetic resonance imaging (MRI) of the right hip one year prior were normal. Lumbar spine imaging performed approximately six months earlier revealed normal radiographs, whereas MRI demonstrated mild disc bulges measuring 1 mm in the anteroposterior dimension, without evidence of nerve root impingement. Her first pediatric rheumatologist had ordered serological tests for arthritis and acute inflammatory markers, which were normal, and recommended repeating the lumbar MRI, but had not made a specific diagnosis.

On presentation to the chiropractor, she appeared well, but the lumbar range of motion was limited and reproduced her pain. Sacroiliac joint provocation tests (e.g., thigh thrust, sacroiliac joint compression) were painful bilaterally (Table 1). The neurologic examination, including reflexes, strength, and sensation, was normal. Gait was normal, and there was no evidence of incoordination. Given the chronicity of symptoms, previous therapies to date, morning stiffness, provocative tests, and lack of lasting relief from prior treatments, the chiropractor considered a broad differential diagnosis, including pelvic pathology such as endometriosis, as well as sacroiliitis and myofascial pain. Lyme disease was also considered, given the endemicity of the area, outdoor exposure, and unexplained joint and muscle pain. Upon review of her prior normal laboratory results, the chiropractor ordered further tests, repeating previous ones and adding new ones to support the differential diagnosis (Table 2). Based on the examination findings and suspicion for sacroiliac joint and/or pelvic involvement, the chiropractor also ordered a pelvic MRI approximately one month after the initial chiropractic visit.

**Table 1:** Physical examination tests.

Tests	Results	Interpretation
Lumbar range of motion	Limited in all planes with pain reproduction	Nonspecific finding associated with pain
Thigh thrust	Painful bilaterally	Potential sacroiliac joint involvement
Sacral thrust	Painful bilaterally	Potential sacroiliac joint involvement
Sacroiliac joint compression	Painful bilaterally	Potential sacroiliac joint involvement
Lower extremity reflexes, strength, and sensation	Normal	Unlikely to reflect nerve root or intraspinal etiology

**Table 2:** Laboratory test results with corresponding reference ranges. *Tests reordered by chiropractor, already ordered by primary care and/or rheumatology in prior year(s), are indicated by an asterisk. IFA: immunofluorescence assay; HLA-B27: human leukocyte antigen B27; CCP: cyclic citrullinated peptide; OD: optical density

Laboratory tests	Results	Reference range
Lyme antibodies, screen	≤0.90 OD index value	≤0.90 OD index value
Rheumatoid factor*	<10 IU/mL	<14 IU/mL
Antinuclear antibody screen, IFA*	Negative	Negative
C-reactive protein*	<3.0 mg/L	<8.0 mg/L
Erythrocyte sedimentation rate*	2 mm/h	≤20 mm/h
HLA-B27 typing	Negative	Negative
Anti-CCP antibody IgG qualitative	Negative	Negative
Anti-CCP antibody, IgG	<15 U/mL	<20 U/mL
Vitamin D 25 hydroxy	41.7 ng/mL	31.0-80.0 ng/mL
Ferritin	16.7 ng/mL	14.7-205.1 ng/mL
Iron	22 µg/dL	41-186 µg/dL
Total iron-binding capacity	504 µg/dL	232-386 µg/dL

With consent from the patient and her mother, the chiropractor also initiated a trial of care while awaiting test results and imaging. Visits were scheduled every two weeks with the goal of providing symptomatic relief. Across four visits, interventions included soft-tissue manipulation (i.e., instrument-assisted soft-tissue manipulation) and dry needling, both targeting the lumbar erector spinae, and spinal and sacroiliac joint manipulation, which offered only transient improvement. A gap in care then occurred as the patient developed infectious mononucleosis, and the patient underwent pelvic MRI 10 weeks after the chiropractic encounter.

**Figure 1:**
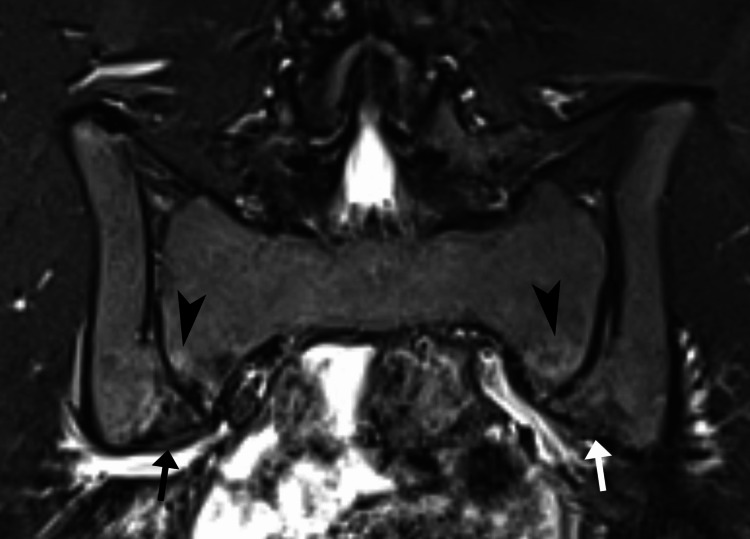
Coronal short tau inversion recovery magnetic resonance image illustrating bilateral sacroiliac edema. Hyperintense signal is present surrounding the inferior third of both sacroiliac joints (arrowheads), consistent with bilateral subchondral bone marrow edema, and hypointense regions suggestive of bony sclerosis (black arrow indicating right side; white arrow indicating left side).

**Figure 2:**
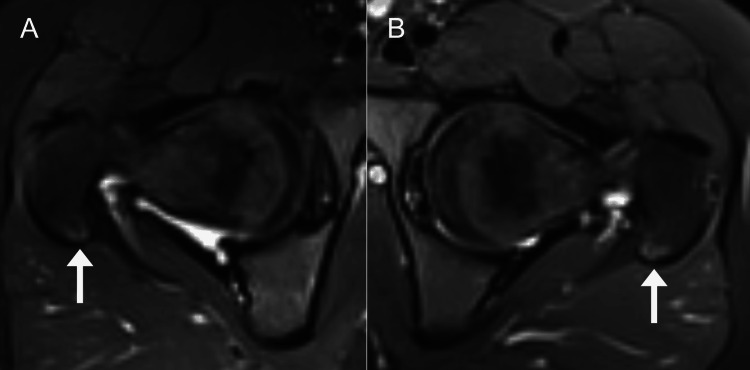
Axial short tau inversion recovery magnetic resonance image illustrating bilateral greater trochanter edema (arrows). A hyperintense signal is evident in the posterior-superior greater trochanter of both the right (A) and left (B) hip.

The pelvic MRI revealed bilateral sacroiliac joint sclerosis, fatty metaplasia, and subchondral edema in the inferior third of both sacroiliac joints (Figure 1), as well as mild bone marrow edema at the gluteus medius tendon insertions of the greater trochanters bilaterally (Figures 2A, 2B). There was no evidence of ovarian cysts or endometriosis, reducing concern for gynecologic causes of pain. In the context of chronic back pain beginning in early adolescence, prolonged morning stiffness, positive sacroiliac joint provocation tests, and limited response to prior therapies, the sacroiliac lesions raised concern for inflammatory sacroiliitis. Based on this combination of features, the chiropractor discussed the results with the patient and her mother and prepared a referral note summarizing the history, examination, and imaging findings directed to a pediatric rheumatologist within the same healthcare system. This clinician was selected for their specific expertise in ankylosing spondylitis and juvenile arthritis.

Approximately three months after the initial chiropractic visit, the pediatric rheumatologist noted bilateral sacroiliac joint tenderness and positive provocation tests (flexion, abduction, and external rotation; Gaenslen’s test) on examination. There was also tenderness over the greater trochanters, consistent with enthesitis, given the symptoms and MRI findings. After reviewing the pelvic MRI by a musculoskeletal radiologist, the MRI findings of subchondral sacroiliac joint edema and sclerosis were interpreted as compatible with sacroiliitis when mechanical causes, such as sacroiliac joint dysfunction, were excluded. After consultation with a pediatric orthopedist to rule out structural or mechanical explanations, the rheumatologist diagnosed bilateral sacroiliitis consistent with juvenile-onset axSpA, specifically juvenile ankylosing spondylitis. Laboratory screening, including tuberculosis testing and blood counts, was obtained prior to initiating a tumor necrosis factor alpha inhibitor. Treatment with this medication, specifically etanercept, started approximately one month later.

**Figure 3:**
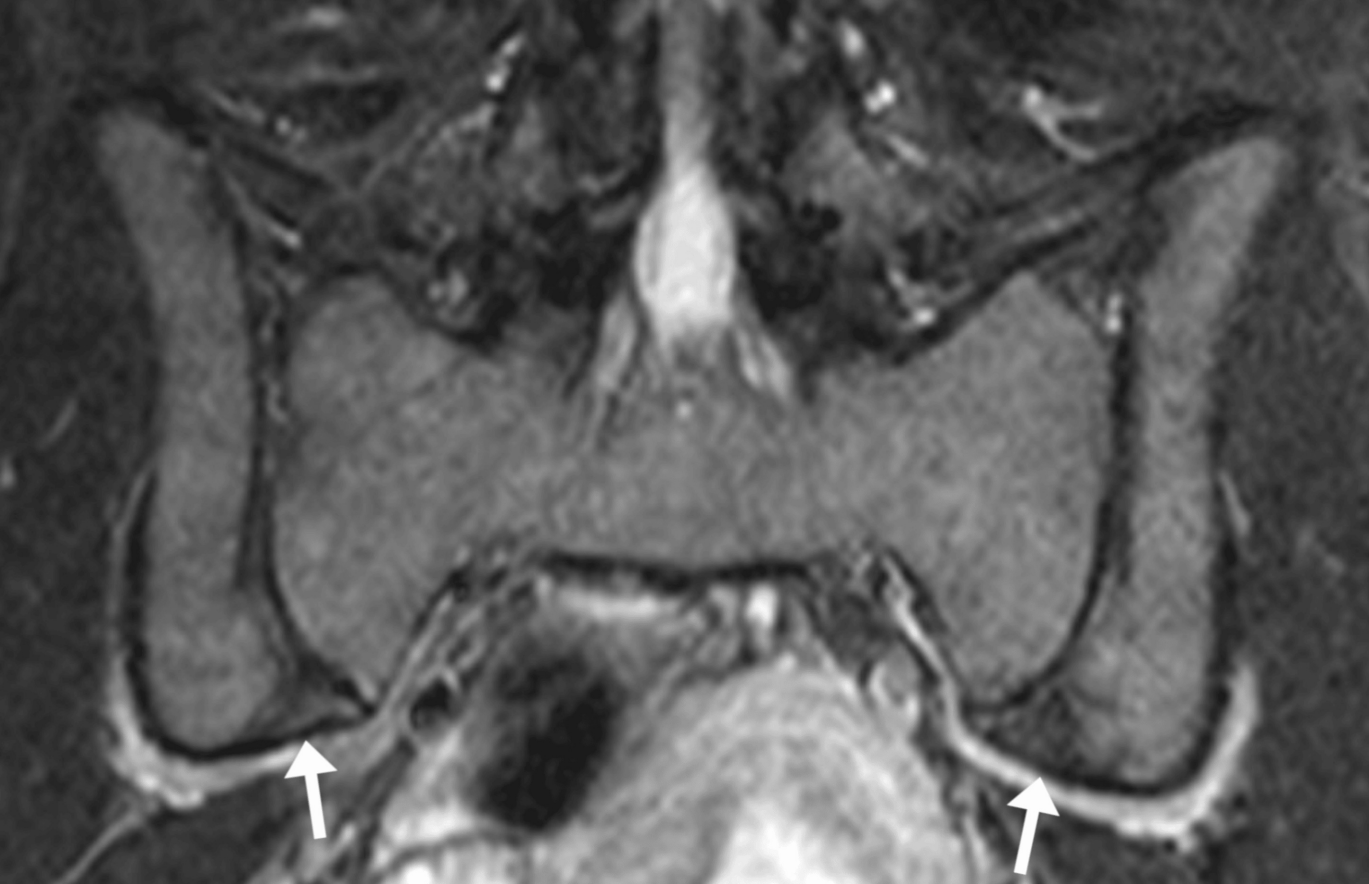
Follow-up coronal short tau inversion recovery magnetic resonance image illustrating improvements. While some evidence of sacroiliac joint sclerosis remains evident (arrows), there is no longer any evidence of hyperintense signals that would correspond to subchondral bone marrow edema.

**Figure 4:**
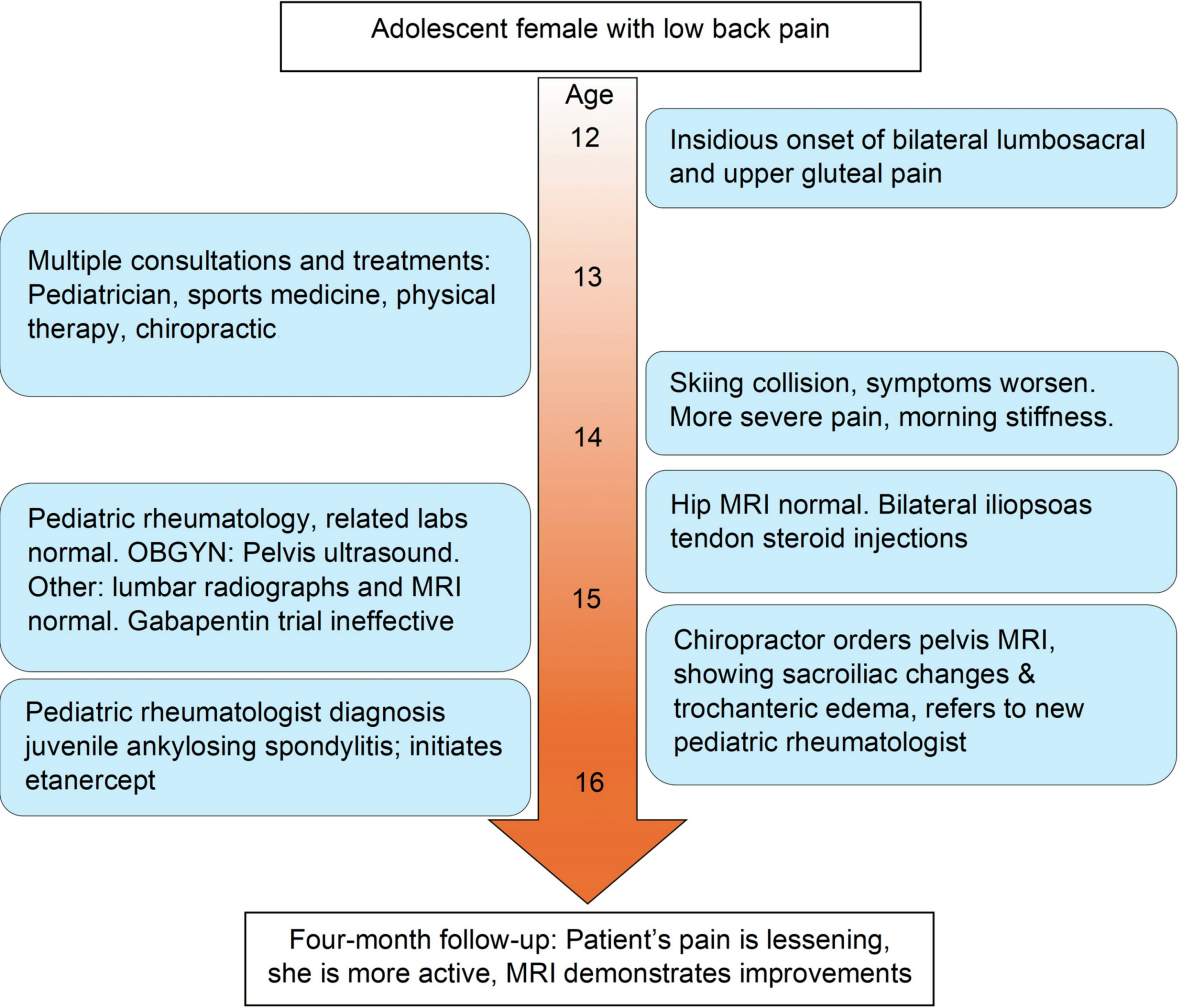
Timeline of the case. OBGYN: obstetrician-gynecologist; MRI: magnetic resonance imaging

Three months after initiating biologic therapy, the patient reported partial improvement, with pain reduced to approximately 5/10 (numeric pain rating scale) and improved daily function. She was now able to walk four miles daily but continued to avoid high-impact activities such as running. Follow-up imaging was planned later in the year. Following additional follow-up, the chiropractor recommended low-impact exercises, such as walking and weight resistance machines, while the rheumatologist referred the patient for an ophthalmological examination for screening purposes. Four months after initiating biologic therapy, the patient underwent a repeat MRI of the pelvis, which demonstrated radiological improvement in the sacroiliac joints compared with the previous study (Figure 3). Additionally, the previously identified greater trochanter edema was no longer evident. The patient continued her walking regimen at three to five miles per day. A timeline of the case is presented in Figure 4.

## Discussion

This case describes a 15-year-old female with a three-year history of insidious bilateral lumbosacral and upper gluteal pain who was ultimately diagnosed with juvenile-onset ankylosing spondylitis by a pediatric rheumatologist after visiting a chiropractor and undergoing pelvic MRI.

A three-year diagnostic delay in this case aligns with literature describing median delays of 5.5 years in pediatric-onset axSpA and two to six years in adult populations [3,4]. Several factors likely contributed to the diagnostic delay in the present case. First, there was over-reliance on lumbar spine imaging and incidental findings of minimal disc bulges. This led to symptoms being attributed to a mechanical etiology and prompted recommendations for repeat lumbar MRI rather than targeted pelvic imaging. Normal inflammatory markers, including C-reactive protein and erythrocyte sedimentation rate, may also have contributed, as these results can create false reassurance, though they are frequently normal in juvenile axSpA and do not exclude the disease [2,11]. In addition, the patient’s early hip symptoms were initially attributed to snapping hip syndrome, which may have obscured an underlying inflammatory cause, although the exact timeline of hip enthesitis relative to sacroiliac joint involvement remains uncertain.

According to the recently validated American College of Rheumatology classification criteria for juvenile axSpA, the patient exceeded the ≥55 point threshold, based on bilateral sacroiliac MRI changes, sacral and buttock pain, insidious onset, prolonged morning stiffness, and persistent symptoms lasting more than 12 weeks [12]. In its initial validation, the criteria demonstrated high specificity (97.5%) and positive predictive value (97.4%) compared with expert diagnosis of axSpA, and demonstrated greater overall accuracy compared with earlier classification systems, including those developed by the assessment of SpondyloArthritis International Society, the International League of Associations for Rheumatology, and the European Spondylarthropathy Study Group [12]. Accordingly, in the current case, the patient’s classification strongly supports the clinical diagnosis of juvenile axSpA.

This case corroborates existing research indicating that chiropractors may encounter undiagnosed axSpA cases [9,13]. One US claims-based study of 97,469 adults with axSpA found that 64.7% had visited a chiropractor for back pain prior to diagnosis, although the study did not specify whether the chiropractors played a role in clarifying the diagnosis [13]. Chiropractors should be aware of inflammatory back pain patterns and consider appropriate laboratory testing, imaging, and or referral to rheumatology when clinical suspicion exists. While radiographs are a reasonable first step, sacroiliac joints often appear normal in juvenile axSpA, whereas MRI is more sensitive to detect subtle and early changes of sacroiliitis, such as bone marrow edema surrounding the sacroiliac joint [2]. In the present case, the combination of positive sacroiliac joint provocation tests, characteristic symptom patterns, and poor response to conservative care prompted a targeted MRI that was used to support a clinical diagnosis of juvenile ankylosing spondylitis.

This study has several limitations. The case represents a single patient experience and may not be generalizable to all presentations of juvenile axSpA. The patient’s gradual improvement to tumor necrosis factor alpha (TNF-α) inhibition was typical and expected for axSpA, as biologic therapies often require several months to achieve maximal benefit, and continued improvement may be expected. However, this case could be strengthened by longer follow-up, patient-reported outcome questionnaires, and serial imaging to further characterize the disease course, treatment response, and related quality-of-life and functional outcomes.

## Conclusions

The present study describes a 15-year-old female with a three-year history of persistent back and hip pain who was ultimately diagnosed with juvenile-onset axSpA after a chiropractor ordered a pelvic MRI and referred her to pediatric rheumatology. As illustrated in the present case, diagnostic delay may have been perpetuated by false reassurance from normal laboratory testing, normal radiographs, and a lack of targeted MRI of the sacroiliac joints. Chiropractors should remain alert to pediatric axSpA features, such as prolonged morning stiffness, nocturnal pain, enthesitis or hip pain, and a poor response to conservative care, and consider timely imaging and referral to pediatric rheumatology in suspected cases.
